# Metabolic alterations in the erythrocyte during blood-stage development of the malaria parasite

**DOI:** 10.1186/s12936-020-03174-z

**Published:** 2020-02-27

**Authors:** Shivendra G. Tewari, Russell P. Swift, Jaques Reifman, Sean T. Prigge, Anders Wallqvist

**Affiliations:** 1 Department of Defense Biotechnology High Performance Computing Software Applications Institute, Telemedicine and Advanced Technology Research Center, U.S. Army Medical Research and Development Command, Ft. Detrick, MD USA; 2 The Henry M. Jackson Foundation for the Advancement of Military Medicine, Inc. (HJF), Bethesda, MD USA; 3 Department of Molecular Microbiology and Immunology, Johns Hopkins University, Baltimore, MD USA

**Keywords:** *Plasmodium falciparum*, Host–parasite metabolism, Blood-stage infection, Metabolome, Lysophosphatidylglycerol, Polyunsaturated fatty acids

## Abstract

**Background:**

Human blood cells (erythrocytes) serve as hosts for the malaria parasite *Plasmodium falciparum* during its 48-h intraerythrocytic developmental cycle (IDC). Established in vitro protocols allow for the study of host–parasite interactions during this phase and, in particular, high-resolution metabolomics can provide a window into host–parasite interactions that support parasite development.

**Methods:**

Uninfected and parasite-infected erythrocyte cultures were maintained at 2% haematocrit for the duration of the IDC, while parasitaemia was maintained at 7% in the infected cultures. The parasite-infected cultures were synchronized to obtain stage-dependent information of parasite development during the IDC. Samples were collected in quadruplicate at six time points from the uninfected and parasite-infected cultures and global metabolomics was used to analyse cell fractions of these cultures.

**Results:**

In uninfected and parasite-infected cultures during the IDC, 501 intracellular metabolites, including 223 lipid metabolites, were successfully quantified. Of these, 19 distinct metabolites were present only in the parasite-infected culture, 10 of which increased to twofold in abundance during the IDC. This work quantified approximately five times the metabolites measured in previous studies of similar research scope, which allowed for more detailed analyses. Enrichment in lipid metabolism pathways exhibited a time-dependent association with different classes of lipids during the IDC. Specifically, enrichment occurred in sphingolipids at the earlier stages, and subsequently in lysophospholipid and phospholipid metabolites at the intermediate and end stages of the IDC, respectively. In addition, there was an accumulation of 18-, 20-, and 22-carbon polyunsaturated fatty acids, which produce eicosanoids and promote gametocytogenesis in infected erythrocyte cultures.

**Conclusions:**

The current study revealed a number of heretofore unidentified metabolic components of the host–parasite system, which the parasite may exploit in a time-dependent manner to grow over the course of its development in the blood stage. Notably, the analyses identified components, such as precursors of immunomodulatory molecules, stage-dependent lipid dynamics, and metabolites, unique to parasite-infected cultures. These conclusions are reinforced by the metabolic alterations that were characterized during the IDC, which were in close agreement with those known from previous studies of blood-stage infection.

## Background

In 2017, there were 219 million cases of malaria worldwide and the ten most burdened African countries saw an estimated 3.5 million more cases than in 2016 [1]. *Plasmodium falciparum* is responsible for 99.7% of all malaria cases in the World Health Organization (WHO) African region, which accounted for 93% of all malarial deaths in 2017 [1]. During the symptomatic stage of malaria, *P. falciparum* resides in human blood cells (erythrocytes) as it multiplies asexually during the 48-h intraerythrocytic developmental cycle (IDC) [2]. The human erythrocyte is also the main conduit for providing *P. falciparum* with essential nutrients during its development during the IDC [3]. While the interactions of the parasite with its host, the human erythrocyte, have been studied for well over a century, much remains to be characterized and discovered. For example, although parasite-infected erythrocytes rapidly sequester arginine from the culture medium under in vitro conditions [4], the relevance of this to parasite development is unclear. In recent years, high-resolution metabolomic methods have been employed to improve the understanding of host–parasite interactions, with the aim of ultimately identifying novel treatments and diagnostic strategies [5–7].

Here, synchronous cultures of the *P. falciparum* parasite were generated in human erythrocytes and globally targeted mass spectrometry was employed to quantify metabolic changes in uninfected and parasite-infected erythrocyte cultures during the IDC. Specifically, the aim of the study was to characterize parasite development during this phase at six equally spaced time points that roughly covered its early, intermediate, and late stages. Although previous studies [4, 8] have also examined metabolomic data at several time points, their methods primarily quantified metabolites involved in carbohydrate, amino acid, and nucleotide metabolism. Yet, during the IDC, *P. falciparum* also synthesizes lipids that are essential not only for membrane biogenesis but also for lipid-dependent signaling or trafficking processes [9]. Therefore, extraction and detection methods that reproducibly quantify ~ 850 metabolites (of which roughly half are lipids) across different cohorts and disease conditions were employed to track these lipids.

To delineate *P. falciparum* metabolism using metabolomic data collected during the IDC, a series of computational methods were used and key global, pathway-level, and stage-specific metabolites were identified. This revealed time-dependent and time-independent alterations in nucleotide, lipid, and carbohydrate metabolites, which were associated with parasite development. Analyses of uninfected and parasite-infected cultures showed significant enrichment in metabolites associated with lipid synthesis. Specifically, lipids showed significant enrichment, which was associated with increased abundance of several polyunsaturated fatty acids (PUFAs) in infected cultures relative to uninfected cultures. Stage-dependent analyses revealed dynamic alterations in the abundance of lysophosphatidylglycerol (LPG) metabolites. The results provide an initial explanation of how LPG metabolites could contribute to *P. falciparum* development during the IDC.

## Methods

### Parasite culture, purification of erythrocytes, and sample collection

*Plasmodium falciparum* NF54 parasites (generously provided by David Fidock, Columbia University) were propagated in O-positive human erythrocytes at 2% haematocrit in gassed flasks (94% N_2_, 3% O_2_, and 3% CO_2_) at 37 °C. Human erythrocytes were obtained as part of an institutional review board-approved phlebotomy protocol (NA_00019050) and used within 2 days after isolation. The infected erythrocytes were maintained in Roswell Park Memorial Institute (RPMI) 1640 medium (Gibco, Gaithersburg, MD) and supplemented with 20 mM HEPES, 12.5 µg/mL hypoxanthine, 0.3% sodium bicarbonate, 25 µg/mL gentamicin, 0.5 µM R-lipoic acid, and 0.5% AlbuMAX II (Life Technologies Inc., Carlsbad, CA). Erythrocytes depleted of white blood cells (WBCs) were used for the parasite culture. First, the buffy coat was removed following two rounds of density gradient centrifugation. The enriched erythrocytes were then overlaid on a 60% Percoll solution and centrifuged at 1500×*g* for 30 min. After removal of WBCs from the interface, the pelleted erythrocytes were carefully collected and washed several times in RPMI-1640 before the haematocrit was adjusted to 50%.

To generate synchronized parasites, the cultures were passed through magnetically activated cell sorting (MACS) columns (Miltenyi Biotec, Auburn, CA) and purified, every 44–48 h for 4 days before the initiation of the experiment. Giemsa-stained blood smears and light microscopy were used to monitor parasitaemia and synchronicity. Additionally, immediately before sample collection, the absence of contaminating mycoplasma was confirmed by a polymerase chain reaction, using primers specific for the gene encoding 16S ribosomal RNA (5′-GGAGCAAACAGGATTAGATACCC and 5′-CACCATCTGTCACTCTGTTAACC).

Before data collection, a synchronized parasite culture (300 mL) was passed through a MACS column in four 75-mL volumes, each eluted with 20 mL of culture medium. The eluates were pooled and adjusted to a total culture volume of 300 mL at 2% haematocrit using leukodepleted blood, and then the culture was divided into four 75-mL replicate flasks. Four flasks containing 50 mL of media with uninfected leukodepleted erythrocytes at 2% haematocrit provided the control group samples for the metabolomic analysis. MACS purification resulted in elution of late-stage trophozoites. The cultures were regularly observed via blood smear until 0–2 h after merozoite invasion of the erythrocytes (final parasitaemia of 7%), at which point the culture media in all flasks was replaced with fresh media (time 0 for this experiment).

Cells were harvested from the four test flasks and the four control flasks via collection of at least 7 mL of culture from each flask. Then, after centrifugation of the tubes at 1500×*g* for 5 min to pellet the cells, followed by aspiration of the media, 100 µL of the test- or control-cell pellets was transferred to 1.5-mL tubes, which were flash frozen in an ethanol/dry-ice bath and stored at − 80 °C for subsequent metabolomic analysis. This procedure was repeated at the following times during the IDC: 0, 8, 16, 24, 32, and 40 h. Finally, quadruplicate samples were sent to Metabolon, Inc. (Durham, NC) for metabolite analysis.

### Global metabolomic profiling of intracellular data

Staff at Metabolon Inc. inventoried the samples and then immediately stored them at − 80 °C. The protocol for metabolomic profiling involved the following steps: (1) precipitate the proteins with methanol under vigorous shaking for 2 min using a GenoGrinder 2000 (Glen Mills Inc., Clifton, NJ), (2) centrifuge the precipitate to remove the proteins, dissociate small molecules bound to the proteins or trapped in the precipitated protein matrix, and recover chemically diverse metabolites, and (3) divide the resulting extract into five fractions [two for analysis by two separate reverse-phase (RP) ultrahigh-performance liquid chromatography (UPLC) tandem mass spectrometry (MS/MS) methods with positive ion mode electrospray ionization (ESI), one for analysis by RP/UPLC–MS/MS with negative ion mode ESI, one for analysis by hydrophilic-interaction chromatography (HILIC) UPLC–MS/MS with negative ion mode ESI, and one for use as a backup sample]. All methods involved the use of a Waters ACQUITY UPLC system (Waters Corp., Milford, MA) and a Q-Exactive high resolution/accurate mass spectrometer (Thermo Fisher Scientific, Hampton, NH) interfaced with a heated electrospray ionization (HESI-II) source and Orbitrap mass analyser operated at 35,000 mass resolution.

Based on the profiling results provided by Metabolon Inc., a total of 501 metabolites of known identity (designated metabolites in the Metabolon© library) were quantified. The raw data were normalized by the Bradford protein concentration of each sample and then any missing value of a metabolite was imputed with its minimum observed value across all samples.

### Global analysis of the data

The intracellular metabolomic data from uninfected (uRBC) and parasite-infected (iRBC) cultures were used as input to the ‘clustergram’ function built into MATLAB^®^. Ward’s hierarchical clustering method and the Euclidean distance metric were used to cluster metabolites with similar temporal profiles in the uRBC and iRBC cultures. Quantile normalization was performed on the filtered data before visualizing the data in the form of a heat map. Principal component analysis (PCA) was performed on the entire data set to identify any separation between the uRBC and iRBC cultures along three principal axes. This was achieved with the use of the ‘pca’ function built into MATLAB^®^.

Fisher’s exact test was used to determine whether a cluster was enriched in a specific metabolite class. Specifically, the following contingency table was computed for a given cluster:

$$ \left[ {\begin{array}{*{20}c} {{\text{N}}_{\text{c}} } & {{\text{N}}_{\text{c}}^{{\prime }} } \\ {{\text{N}}_{\text{d}} } & {{\text{N}}_{\text{d}}^{{\prime }} } \\ \end{array} } \right] $$

where ${\text{N}}_{\text{c}}$ and ${\text{N}}_{\text{d}}$ denote the number of metabolites that belong to metabolite class ‘K’ in cluster ‘C’ and in the entire data set, respectively, and ${\text{N}}_{\text{c}}^{{\prime }}$ and ${\text{N}}_{\text{d}}^{{\prime }}$ represent the number of metabolites that do not belong to class K in cluster C and in the entire data set, respectively. The ‘fishertest’ function built into MATLAB^®^ was used to test the null hypothesis that there was no nonrandom association between metabolite class K and cluster C. If the test rejected the null hypothesis at the 5% significance level, cluster C was deemed to be enriched in metabolite class K.

The average fold change in metabolites detected in the uRBC and iRBC cultures was computed to identify metabolites that were significantly altered during the IDC. To this end, the ‘bootstrp’ function built into MATLAB^®^ was used to generate 10,000 bootstrap samples for each metabolite from four replicate measurements of abundance at each time point. The fold change was then computed according to the following equation:

1 $$ \begin{array}{*{20}c} { {\text{F}}_{\text{i}} = \frac{{{\bar{\text{m}}}_{\text{iRBC}} }}{{{\bar{\text{m}}}_{\text{uRBC}} }} } \\ \end{array} $$

where F_i_ denotes the fold change in a metabolite ‘m’ for the ith bootstrap sample, and ${\bar{\text{m}}}_{\text{iRBC}}$ and ${\bar{\text{m}}}_{\text{uRBC}}$ represent the average abundance levels of the metabolite in the iRBC and uRBC cultures, respectively, where abundance is averaged across all replicates and time points. To obtain the average and standard deviation of the fold change in a metabolite during the IDC, the average and standard deviation of F_i_ were computed across all samples. To obtain the average fold change of a metabolite at a given time point, the same procedure was used, but with F_i_ from the abundance measurements for that time point.

### Pathway enrichment and statistical analyses

To gain mechanistic insights into the altered metabolic pathways, quantitative pathway enrichment analysis (QSEA) was performed using MetaboAnalyst [10] on the processed intracellular metabolomic data from uRBC and iRBC cultures. QSEA identifies associations between metabolite sets and disease conditions, such as parasite infection. The MetaboAnalyst [10] Web tool requires Human Metabolome Database (HMDB) identifiers as input to perform QSEA. Metabolite sets related to human metabolism, which contained at least five metabolites per set, were obtained from the small molecule pathway database (SMPDB). Starting with the SMPDB library [11] of normal human metabolic pathways, KEGG pathway annotations [12] were used to further classify the SMPDB metabolic pathways into six major pathways. Before the analysis, the data were grouped into three different IDC periods, i.e., 0–8, 16–24, and 32–40 h, to characterize stage-relevant parasite development and to increase the sample size for detecting statistically significant differences within each group [13].

To test for infection-specific alterations in metabolites, for each of the 501 metabolites, a two-way analysis of variance (ANOVA) was performed for each IDC stage (early, intermediate, and late), with time point (0 and 8 h, 16 and 24 h, or 32 and 40 h) and infection status (infected or uninfected) as the between-group factors. Prior to the ANOVA, the data were log (base 2)-transformed to make them normally distributed [14]. The ‘anova2’ function built into MATLAB was used to analyse abundance levels for each metabolite to test the degree to which they differed as a function of the IDC period, infection status, and their interaction, at a significance criterion of *p* ≤ 0.05. At each of the three IDC stages, a metabolite that showed a significant interaction between time point and infection status was deemed as an infection-specific metabolite, because this term would capture any change in the difference in the abundance of such a metabolite between the iRBC and uRBC cultures over time. Lastly, the ‘mafdr’ function built into MATLAB was used to implement Storey’s method [15] to estimate the false discovery rate for multiple hypothesis testing. A significantly altered metabolite was rejected if the false discovery rate was 10% or greater (i.e., *q* ≥ 0.10).

## Results

### Metabolomics of blood-stage malaria parasites

**Fig. 1:**
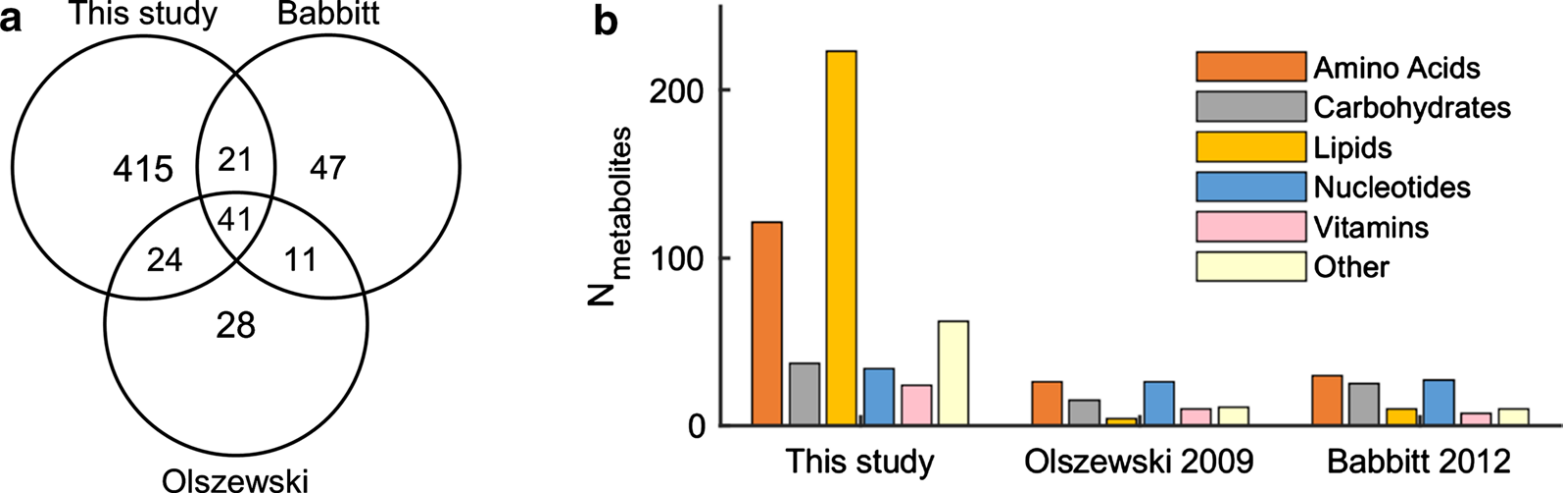
Comparison of metabolite coverage across this study and studies by Babbitt et al. [8] and Olszewski et al. [4]. **a** Venn diagram showing overlap of metabolites between the three studies. Relative to the other two studies, which quantified comparable numbers of metabolites during the intraerythrocytic developmental cycle (IDC), this study quantified roughly five times more metabolites. **b** Metabolites detected in the three studies (N_metabolites_) mapped onto five major metabolic pathways. In contrast to the previous studies, which quantified ~ 100 metabolites during the IDC, this study quantified over 200 lipid metabolites and more than 100 amino acid metabolites. “Other” denotes metabolites that do not belong to the five major metabolic pathways

Recent years have seen a surge in the use of metabolomics to probe biological and physiological systems of interest. Specifically, methods have been developed to quench the metabolism of parasite-infected erythrocytes and study their metabolite extracts [6]. Metabolic profiling approaches are used to characterize blood-stage parasite development [4, 16], discover biomarkers [17, 18], and identify novel therapeutic targets [19]. Table 1 lists studies that have examined the blood stage of malaria parasites using metabolomics. Of note are two studies [4, 8] that characterized more than 100 metabolites in synchronous cultures of *P. falciparum* at several time points during the IDC. Figure 1 shows a comparison of the metabolite coverage for these studies and the current study, as well as the overlap of metabolites between the studies. Whereas the number of nucleotide metabolites was comparable across the three studies, that of the lipid metabolites was many-fold higher in the current study (Fig. 1b). The extraction methods and mass spectrometry platforms used in previous studies precluded them from detecting a large number of lipid metabolites [4, 8]. Several recent studies [6, 23, 24] quantified more metabolites than in the studies by Babbitt et al. [8] or Olszewski et al. [4]. However, they either did not examine all parasite stages or did not include matched uninfected erythrocyte cultures during the IDC (see Table 1). Therefore, it was not possible to comprehensively compare the current study with the recent studies. In the following sections, a global analysis of the collected data will be presented first. Subsequently, the metabolic changes that characterize parasite development during the IDC will be examined.

**Table 1:** Metabolomic data obtained from blood-stage parasites

N_metabolites_	N_time_	*n*^a^	Source of data	Year	References
*Obtained at multiple time points from uninfected and infected erythrocytes*					
104	7	3	Uninfected and parasite-infected synchronous cultures of erythrocytes	2009	[4]
120	10	2	Uninfected and parasite-infected synchronous cultures of erythrocytes	2012	[8]
501	6	4	Uninfected and parasite-infected synchronous cultures of erythrocytes	–	This study
*Other relevant data obtained during the IDC*					
52	1	13	Saponin-treated synchronous parasite culture	2009	[16]
104	1	4	Uninfected and parasite-infected asynchronous cultures of erythrocytes (absence/presence of streptolysin O)	2013	[20]
76	5^b^	6	Parasite-infected synchronous cultures of erythrocytes	2016	[21]
113	1	3	Parasite-infected synchronous cultures of erythrocytes	2016	[22]
460	1	4	Parasite-infected synchronous cultures of erythrocytes	2016	[6]
583	1	3^c^	Uninfected and parasite-infected synchronous cultures of erythrocytes	2017	[23]
297	1	5	Saponin-treated synchronous parasite culture	2018	[24]

*N*_*metabolites*_ number of metabolites quantified, *N*_*time*_ number of time points sampled during the intraerythrocytic developmental cycle (IDC)

^a^Number of replicates at each time point

^b^Time points cover the first 6 h after treatment by a drug

^c^Data from uninfected erythrocytes do not have a replicate

### Global metabolomic profiling of uRBC and iRBC cultures

Five hundred and one metabolites present in either uRBC or iRBC cultures were quantified. Of these, 481 were present in both cultures and 20 were unique to iRBC cultures. An analysis of the former set is presented first, followed by an analysis of the iRBC-specific metabolites in the subsection titled “Metabolites uniquely associated with parasite infection” of the paper. Of the 501 metabolites, 333 and 261 were annotated in the HMDB [25] and *Plasmodium* [26] metabolite databases, respectively, with 9 unique to the latter. A total of 159 metabolites were not annotated in either database, although it was possible to assign 2 of them to KEGG identifiers. Thirty-one percent (157/501) of the metabolites could not be linked back to any of these databases (Additional file 1), indicating a general lack of specific pathway information for these compounds. Of these unannotated metabolites, 79% (124/157) and 8% (12/157) belonged to pathways involved in lipid metabolism and amino acid metabolism, respectively. A small percentage (7/157) of the unannotated metabolites belonged to pathways involved in xenobiotic metabolism. Additional files 2 and 3 include lists of all of the metabolites found in the *Plasmodium* metabolite database [26] and Malaria Parasite Metabolic Pathways database [27], respectively.

**Fig. 2:**
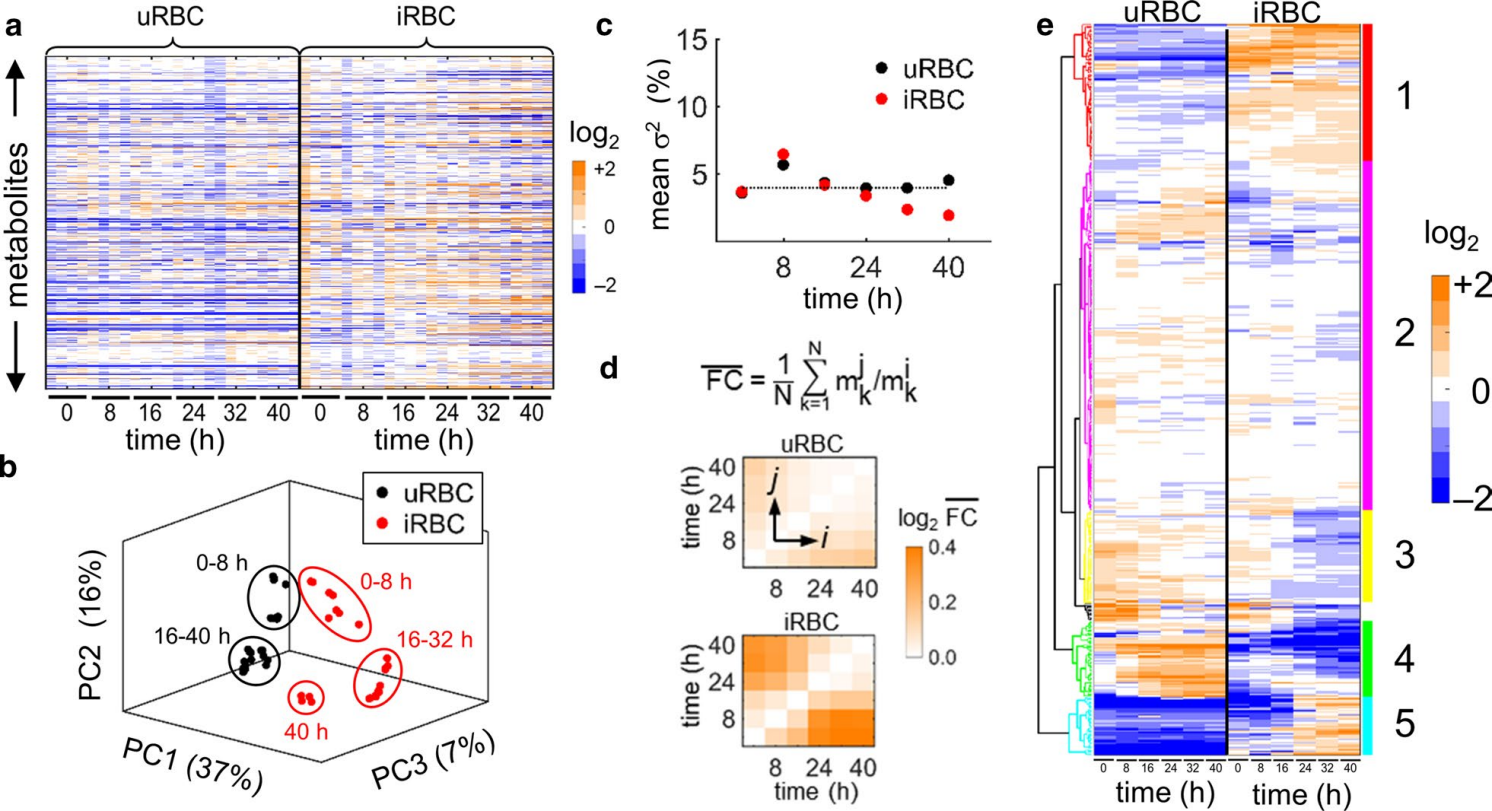
Global metabolomics of uninfected (uRBC) and parasite-infected erythrocyte (iRBC) cultures. **a** Heatmap of metabolite abundances in uRBC and iRBC at 0, 8, 16, 24, 32, and 40 h. Each of the 501 rows represents a distinct metabolite. There are four replicates for each time point. Orange indicates an abundance level of a metabolite greater than the median value, which is computed across uRBCs and iRBCs, whereas blue indicates an abundance level lower than the median. **b** Principal component analysis of metabolomic data from uRBCs (black) and iRBCs (red). The uRBC and iRBC data separated along the first (PC1), second (PC2), and third (PC3) principal components, with the maximum separation occurring between the ellipses labelled ‘16–40 h’ and ‘16–32 h,’ respectively. The uRBC data formed two clusters: 0–8 h and 16–40 h. Ellipses are drawn only to visually highlight uRBC and iRBC data that were clustered together; they do not reflect the confidence intervals of the clusters. The ellipses labelled ‘16–32 h’ and ‘16–40 h’ contain 12 and 16 data points, respectively, although they are not discernible because of overlap among some of the data points. The percentage of the total data variance explained by each principal component is shown in parentheses along each axis. **c** Average variance (σ^2^) of metabolite abundance at a given time point within replicates. First, the variance within replicates is computed for the abundance of a given metabolite and then the average across all metabolites is computed for each time point. The average variance is shown in black for uRBCs and in red for iRBCs. The dotted horizontal line shows the mean of the average variance, which is ~ 4%. **d** The average fold change ($\overline{\text{FC}}$) in metabolite abundance between different time points. The fold change in the kth metabolite at time point ‘*j*’ against time point ‘*i*’ is computed as ${\text{m}}_{\text{k}}^{\text{j}} / {\text{m}}_{\text{k}}^{\text{i}}$, where *i* and *j* are each set to 0, 8, 16, 24, 32, or 40 h. Hence, each element *ij* indicates the average metabolite fold change computed using the dataset at time points *i* and *j*, where N denotes the total number of metabolites. Compared to the average metabolite fold changes in uRBCs, those in iRBCs are more pronounced at all sampled time points. The results are shown on a log_2_ scale. **e** Hierarchical clustering analysis (HCA) of the metabolomic data in **a** after averaging the metabolite abundances among the replicates. The colour scheme and scale are as shown in **a**. Metabolites were clustered based on the Euclidean distance similarity of their temporal profiles. HCA identified five distinct clusters, which are shown in distinct colours with a corresponding number. Generally, within each cluster, metabolites that were downregulated in uRBCs were upregulated in iRBCs and vice versa

Figure 2a shows the individual log_2_ fold-change values for all identified metabolites as measured for each replicate at each time point. Compared to iRBC cultures, which showed increases in fold-change values late in the IDC (32–40 h), uRBC cultures showed fewer metabolite changes over time. To ascertain the overall characteristics and time-dependent alterations of the metabolite data, PCA was performed on the fold-change data in Fig. 2a. Figure 2b shows a clear separation of the data for the two culture systems over time, with the four replicate data points for each time point allowing visualization and assessment of the data spread.

Five ellipses visually separated the data into five clusters (0–8 h, 0–8 h, 16–32 h, 16–40 h, and 40 h), each of which contains either uRBC or iRBC data plotted along the three principal components for the indicated time range. Not all of the replicates are discernible because some data points overlap. For example, the ellipses labelled ‘16–32 h’ and ‘16–40 h’ contain 12 and 16 data points, respectively. Early in the IDC (0–8 h), the data for the two sets of cultures (infected and uninfected) were not identical but similar. It should be noted that the uninfected cultures and the cultures infected with synchronized late-stage parasites were incubated for several hours until ring-stage parasites were observed and sample collection began (*t* = 0). This incubation period and the relatively low metabolic activity of the new ring-stage parasites are likely responsible for the slight separation between uRBC and iRBC cultures along the first principal component (PC1) at 0–8 h. The uRBC cultures underwent metabolic changes over time, as shown by the separation along the second principal component (PC2) between the 0–8 h and 16–40 h time points. The separation between the uRBC and iRBC data along PC1 was greatest late in the IDC (16–32 h), coinciding with the appearance of trophozoites and schizonts in iRBCs. At the last data point (40 h), there was less separation between uRBC and iRBC cultures. That is, the metabolic profile of iRBC cultures corresponding to the late-stage schizogony of the parasite began to resemble that of uRBC cultures at 16–40 h. To ascertain measurement variation, the variance in the data arising from replicate measurements was calculated for both culture systems. Figure 2c shows the average metabolite variance for uRBCs and iRBCs as a function of time of data collection. For both systems, the average relative variance was ~ 4%, with slightly lower variability in iRBC cultures at later time points, presumably because the abundant metabolites produced by late-stage parasites were quantified with more precision. Overall, the variability at all time points was low, demonstrating the robustness of the culture and metabolomics methods. Thus, in the following analysis, the data were averaged over replicates.

To further gauge time-dependent changes in overall metabolic activity, the average fold change in metabolite abundance ($\overline{\text{FC}}$) was calculated between each pair of time points. Figure 2d shows this quantity for both culture systems, where the diagonal elements are zero (much as in a correlation matrix) because the metabolite abundance at each time point is compared to itself. For uRBCs, the changes were modest (Fig. 2d, top), as reflected in the subtle changes between the early (0–8 h) and late time points (Fig. 2b, black clusters). In contrast, for iRBCs the changes were greater (Fig. 2d, bottom), as reflected in the pronounced changes between the early and late time points (Fig. 2b, red clusters).

To identify the underlying groups of metabolites that drive the changes in metabolite profiles, hierarchical clustering of all 501 metabolites was performed and metabolites that behaved differently in the two culture systems were characterized. Here, the clustering procedure grouped metabolites based on the Euclidean distance similarity of the metabolite fold-change values (see “Methods”). Figure 2e shows that most metabolites could be grouped into five distinct clusters (*Clusters 1*–*5*) based on their wide-ranging changes over time in response to parasite infection. *Cluster 1* consisted mainly of metabolites that were consistently lower in uRBC cultures than in iRBC cultures and higher in iRBC cultures than in uRBC cultures across all time points. *Cluster 3* exhibited a trend opposite to *Cluster 1*, whereas iRBC metabolites in *Clusters 4* and *5* showed marked time-dependent changes associated with IDC progression. *Cluster 2* contained the largest number of metabolites, whose fold-change values were mainly of smaller magnitude and less dependent on time compared to the other clusters.

Cluster enrichment analysis (see “Methods”) was used to ascertain whether specific metabolite classes could be associated with these clusters. *Clusters 1* and *5* were enriched in nucleotide metabolites, *Clusters 2* and *3* in lipid metabolites, and *Cluster 4* in carbohydrate metabolites. This is commensurate with known global metabolic features associated with parasite-infected erythrocyte cultures during the IDC [28–30], i.e., both time-dependent and time-independent production of nucleotide metabolites in *Clusters 5* and *1,* respectively; consumption of carbohydrate metabolites in *Cluster 4*; and an increased depletion of lipids needed to create membranes for parasite progeny in *Cluster 3*.

### Metabolite changes characterizing uninfected and infected erythrocyte cultures

This section describes the quantification of consistent, large metabolite changes between the infected and uninfected cultures—an approach that can potentially identify circulating metabolite biomarkers indicative of malaria infection. Ninety-three metabolites increased (or decreased) twofold in average abundance in iRBC cultures relative to uRBC cultures. Table 2 (top) shows the 15 metabolites that increased most in abundance, with fold changes ranging from ~ 4 (mannose-6-phosphate) to ~ 64 (pipecolate). These belong to *Clusters 1* and *5* (Fig. 2e) and correspond to metabolites that either consistently increased in iRBC cultures (*Cluster 1*), or which showed a clear time-dependent increase in abundance at later time points (*Cluster 5*). Similarly, Table 2 (bottom) shows the 15 metabolites that decreased the most in average abundance, with fold changes ranging from ~ 3 (*N*^6^-carboxymethyllysine) to ~ 33 (fructose-6-phosphate). These metabolites, which all belong to *Cluster 4* (Fig. 2e), were representative of a set of metabolites that consistently decreased in abundance in a stage-dependent manner with IDC progression. Additional files 4 and 5 include the average and temporal fold changes in abundance, respectively, for all 93 metabolites.

**Table 2:** Fold changes in metabolite levels between infected and uninfected erythrocyte cultures

Metabolite	FC_IDC_ (SD)^a^	Pathway	Mass (amu)^b^	Cluster^c^
*15 metabolites showing the greatest increase*				
Pipecolate	63.9 (10.1)	Lysine degradation	130.1	5
Nicotinic acid mononucleotide	33.6 (3.19)	Nicotinate and nicotinamide metabolism	336.0	5
Orotate	19.8 (2.74)	Pyrimidine metabolism	155.0	1
Phosphoethanolamine	13.6 (1.14)	Phospholipid metabolism	140.0	5
Glycerol 2-phosphate	11.0 (1.15)	Glycerolipid metabolism	171.0	5
*N*^1^,*N*^12^-diacetylspermine	7.84 (1.73)	Polyamine metabolism	287.2	1
*N*-acetylserine	7.71 (0.36)	Glycine, serine, and threonine metabolism	146.0	1
Glycerol	7.42 (0.98)	Glycerolipid metabolism	91.0	1
Glycerophosphoglycerol	5.29 (0.23)	Glycerolipid metabolism	245.0	1
Guanine	4.92 (0.89)	Purine metabolism	152.1	1
Putrescine	4.87 (0.83)	Polyamine metabolism	89.1	5
2′-deoxyuridine	4.80 (0.66)	Pyrimidine metabolism	227.1	5
Nicotinate ribonucleoside	4.73 (0.67)	Nicotinate and nicotinamide metabolism	256.1	5
1-stearoyl-GPG (18:0)	4.69 (0.81)	Lysophospholipid	511.3	5
Mannose-6-phosphate	4.29 (1.26)	Fructose, mannose, and galactose metabolism	259.0	5
*15 metabolites showing the greatest decrease*				
*N*^6^-carboxymethyllysine	0.37 (0.04)	Advanced glycation end-product	205.1	4
*S*-lactoylglutathione	0.37 (0.03)	Glutathione metabolism	378.1	4
Pyrraline	0.36 (0.06)	Food component/plant	255.1	4
Dihydroxyacetone phosphate	0.36 (0.03)	Glycolysis, gluconeogenesis, and pyruvate metabolism	169.0	4
3-methylcytidine	0.31 (0.04)	Pyrimidine metabolism	258.1	4
1-linoleoyl-GPC (18:2)	0.30 (0.04)	Lysophospholipid	520.3	4
2-phosphoglycerate	0.29 (0.08)	Glycolysis, gluconeogenesis, and pyruvate metabolism	185.0	4
Ribitol	0.27 (0.03)	Pentose metabolism	151.1	4
Gamma-glutamylglutamate	0.27 (0.03)	Gamma-glutamyl amino acid	277.1	4
Isovalerylglycine	0.26 (0.05)	Leucine, isoleucine, and valine metabolism	158.1	4
1-stearoyl-GPC (18:0)	0.25 (0.04)	Lysophospholipid	524.4	4
1-oleoyl-GPC (18:1)	0.22 (0.04)	Lysophospholipid	522.4	4
1-palmitoyl-GPC (16:0)	0.20 (0.03)	Lysophospholipid	496.3	4
Sedoheptulose-7-phosphate	0.10 (0.01)	Pentose phosphate pathway	289.0	4
Fructose-6-phosphate	0.03 (0.00)	Glycolysis, gluconeogenesis, and pyruvate metabolism	259.0	4

*amu* atomic mass unit, *GPC* glycerophosphocholine, *GPG* glycerophosphoglycerol, *GPI* glycerophosphoinositol, *GPS* glycerophosphoserine, *iRBC* infected erythrocyte, *SD* standard deviation, *uRBC* uninfected erythrocyte

^a^Fold-change (FC_IDC_) values based on a comparison of the average abundance of a metabolite during the IDC in the iRBC culture relative to that in the uRBC culture

^b^Values provided by METABOLON©

^c^Cluster in Fig. 2e containing the metabolite

The largest overall increase in metabolite abundance occurred for pipecolate, a product of lysine catabolism. The increase in pipecolate was associated with the schizont stage (32–40 h) of the iRBC (Additional file 5), in broad agreement with a recent study [18]. Furthermore, pipecolate, which accumulates in the plasma [17] and urine [31, 32] of patients with severe malaria, has been suggested as a candidate clinical biomarker of malaria. Pipecolate is strongly associated with parasite-infected cultures and may be necessary for IDC progression; it is also associated with increased inflammation [33], oxidative stress [34, 35], and epilepsy [36]. The largest reduction in metabolite abundance occurred for fructose-6-phosphate, a product of glycolysis breakdown, reflecting the high glucose-consumption capacity of parasite-infected erythrocytes [29]. Within infected erythrocytes, most of the glucose (60–70%) is incompletely oxidized to lactate and excreted [29]. High lactate dehydrogenase activity, which produces lactate from pyruvate, generates oxidized nicotinamide adenine dinucleotide (NAD^+^) from reduced nicotinamide adenine dinucleotide (NADH) [37]. This process can generate nicotinic acid mononucleotide as a byproduct, which increased 33-fold in abundance (Table 2). Specifically, as NAD^+^ increases, nicotinate-nucleotide adenylyltransferase catalyzes the conversion of NAD^+^ into nicotinic acid mononucleotide [38], resulting in the concomitant accumulation of intraerythrocytic nicotinic acid mononucleotide.

For all metabolites present in both uRBC and iRBC cultures, the observed differences in abundance represent both a RBC response to infection and a parasite component, providing a direct measurement of the intraerythrocytic environment. The largest changes in metabolite abundance occurred mostly for small molecules. Such molecules, which are subject to further biotransformation, may have alternative sources apart from RBC metabolism and, hence, may not be linked readily to observable plasma and urine biomarkers. Interestingly, the data in Table 2 also point to significant changes in a specific lysophospholipid (GPG 18:0), which has not been reported before.

### Global and temporal metabolic activity increase during the IDC

**Fig. 3:**
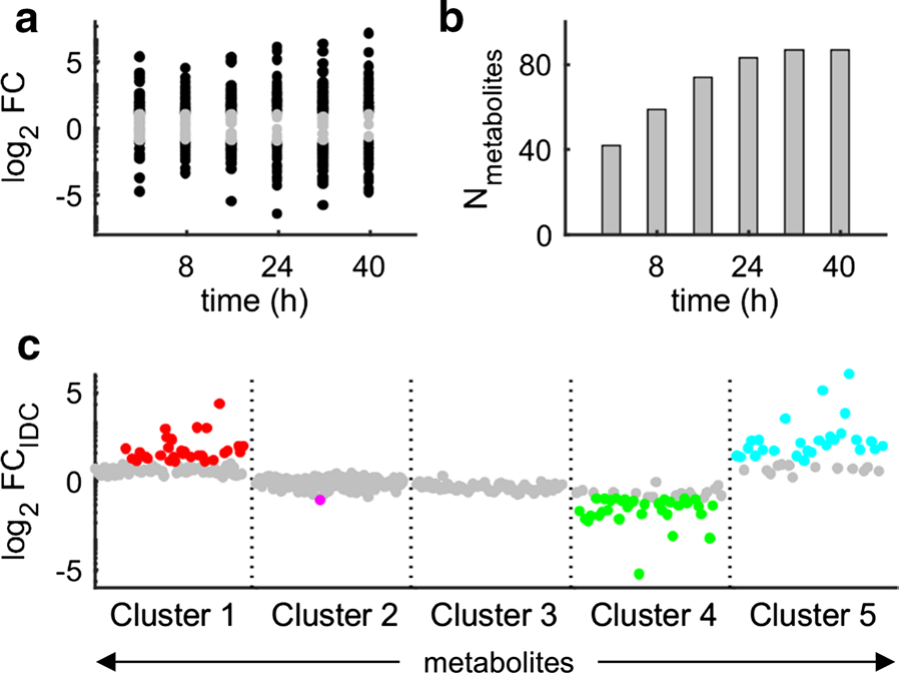
Global and temporal changes in metabolite abundance during the IDC. **a** Temporal fold-change values in significantly altered metabolites. Here, any metabolite that changed (i.e., increased or decreased) twofold or more in abundance was considered as significantly altered. The time-specific fold change was computed as ${\text{m}}_{\text{iRBC}} / {\text{m}}_{\text{uRBC}}$, where m represents the metabolite abundance at 0, 8, 16, 24, 32, or 40 h, and uRBC and iRBC denote uRBC and iRBC cultures, respectively. Fold-change values greater than or equal to two are shown in black, and those smaller than two are shown in grey. **b** The number of metabolites in **a** that changed by twofold or more at the indicated time points. The number increased monotonically with time, suggesting that pronounced metabolic changes occur during the later stages of the IDC. **c** Fold change in the average abundance of metabolites from *Clusters 1*–*5* in Fig. 2e. The fold change in average abundance (FC_IDC_) was computed as ${\bar{\text{m}}}_{\text{iRBC}} / {\bar{\text{m}}}_{\text{uRBC}}$, where ${\bar{\text{m}}}$ represents the average abundance of a metabolite ‘m’ averaged across all time points. Twofold changes in average abundance are shown in red (*Cluster 1*), magenta (*Cluster 2*), green (*Cluster 4*), or cyan (*Cluster 5*). Fold changes of less than two are shown in grey. Although *Cluster 3* showed some temporal changes in metabolites (Fig. 2e), these disappeared when the FC_IDC_ was computed (hence, all markers are grey). *IDC* intraerythrocytic developmental cycle, *iRBC* parasite-infected erythrocyte, *uRBC* uninfected erythrocyte

Infected erythrocyte cultures exhibited continuous and distinct metabolic activity. Figure 3a shows the fold change in the abundance of the top 93 metabolites at the six measured time points (see Additional file 4 for their average fold changes). The magnitude of these fold changes increased from early to later time points. Figure 3b shows that the number of metabolites changing by at least twofold in abundance at each time point increased from 42 (*t* = 0 h) to 87 (*t* = 40 h), suggesting a parasite-induced increase in metabolic activity following infection and a growing divergence of activity between uRBC and iRBC cultures over the course of the IDC. Importantly, although iRBC cultures were less active in the early stages than in the later stages, they were not metabolically inactive.

The metabolites with the highest average fold-change values were concentrated in *Clusters 1*, *4*, and *5* (Fig. 2e). Figure 3c shows the detailed fold change in average abundance of the metabolites from *Clusters 1*–*5,* where the marker colours correspond to those of the annotations for each cluster in Fig. 2e. A fold change of less than two is shown in grey. The average fold change (FC_IDC_) captured sustained alterations in metabolite abundance during the IDC and washed out any transient metabolic alterations [e.g., *Clusters 2* and *3* metabolites in Fig. 2e showed a transient increase in uRBC cultures or a transient decrease in iRBC cultures; however, only one (magenta) of these metabolites crossed the twofold magnitude threshold (Fig. 3c)]. *Clusters 1* and *5* contained metabolites associated with nucleotide metabolism, with 31 (red) and 27 (cyan) increasing to twofold or more in abundance, consistent with the rapid rate of parasitic nucleic acid synthesis [28, 39, 40]. *Cluster 4* contained metabolites associated with carbohydrate metabolism, which in malaria parasites is synonymous with the Embden-Meyerhof-Parnas pathway of glycolysis. Within this cluster, 34 metabolites (green) decreased twofold or more in abundance. This reduction in metabolite abundance is consistent with prior work showing that glucose consumption in *Plasmodium* parasites can increase to as much as 100-fold in iRBC cultures during the advanced stages of the IDC [29].

### Influence of metabolite alterations on metabolic pathways

**Fig. 4:**
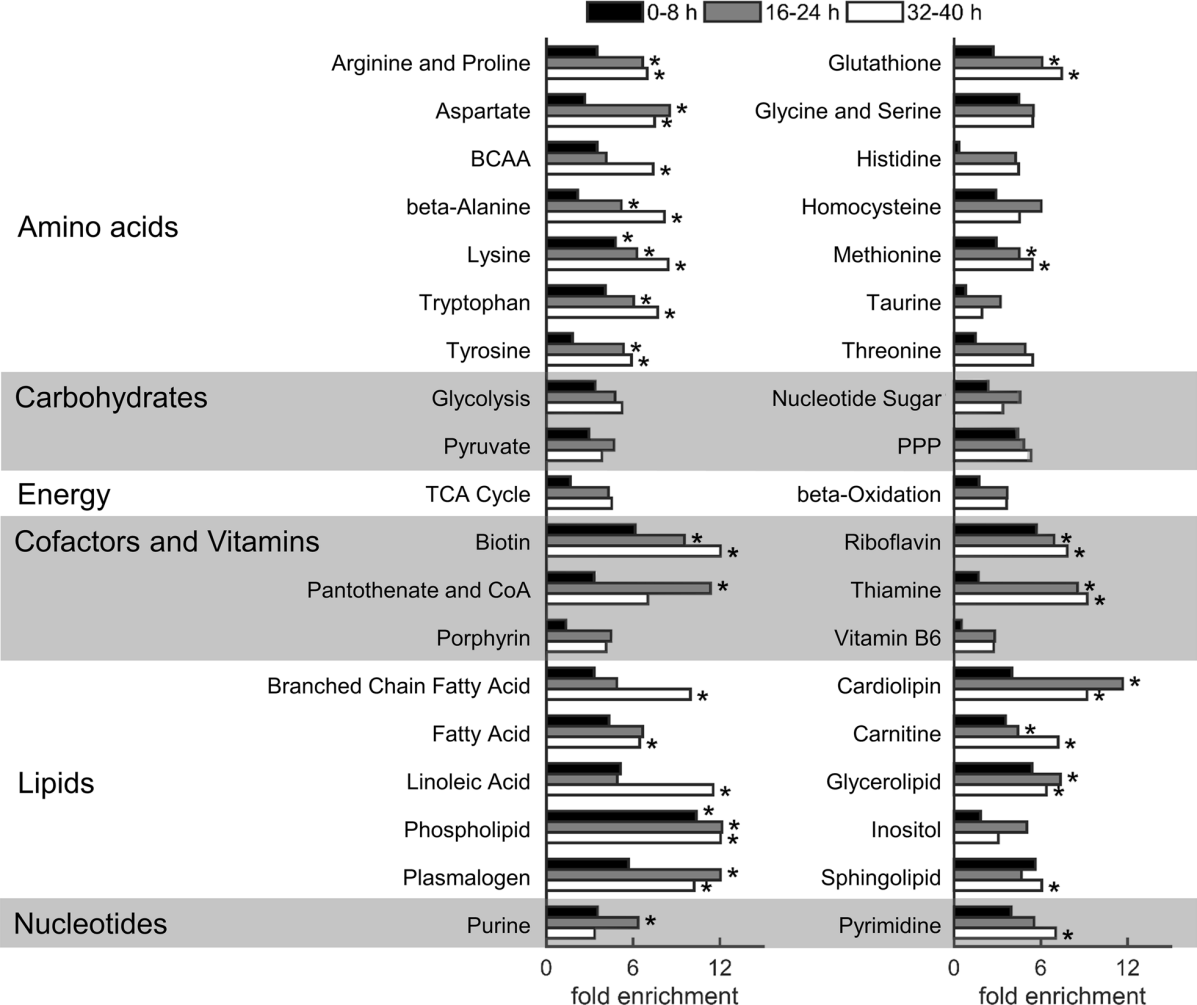
Fold enrichment in human metabolic pathways of parasite-infected erythrocytes at 0–8 h, 16–24 h, and 32–40 h. MetaboAnalyst [10], which takes human metabolome database identifiers as input, was used to compute fold enrichment. Of the pathways in the small molecule pathway database library [11] of normal human metabolic pathways, only those that contained at least five metabolites were used. Asterisks indicate fold-enrichments with an adjusted criterion of *p* ≤ 0.01 [44]. *BCAA* branched-chain amino acid, *CoA* co-enzyme A, *FA* fatty acid, *PPP* pentose phosphate pathway, *TCA* tricarboxylic acid

To identify and quantify the changes among these metabolites associated with parasite development during the IDC, pathway enrichment analysis was further performed on all metabolomic data (Fig. 4). Metabolites of the amino acid class, such as those produced by arginine-proline, aspartate, and glutathione metabolism, showed significant enrichment at intermediate and late time points, consistent with their canonical role in protein synthesis [41] and oxidative stress [42]. This enrichment was associated with haemoglobin degradation, which provides a major source of amino acids for *Plasmodium* parasites [41, 43]. In contrast, lysine degradation was the only amino acid pathway significantly enriched at all time points, suggesting that it continuously functions throughout the entire IDC. None of the carbohydrate or energy metabolism pathways were significantly enriched (Fig. 4). However, the pyruvate metabolism (*p* ≈ 0.03) and tricarboxylic acid (TCA) cycle (*p* ≈ 0.01) pathways both showed enrichment at intermediate time points. This enrichment was associated with l-malate, which increased to 2.5-fold (Additional file 4). Neither *P. falciparum* parasites [45] nor RBCs have a fully-functional TCA cycle [46], which suggests that the parasite produces malate for incorporation into purine nucleotides via oxaloacetate and aspartate [47]. Among nucleotide metabolism pathways, purine showed enrichment (~ sixfold), as did pyrimidine, a metabolite synthesized de novo by *P. falciparum* [48]. Although Fig. 4 shows enrichment in several subordinate pathways of the co-factor and vitamin class, the enrichment in these pathways should not be interpreted as enrichment in any particular co-factor or vitamin metabolite, because it was more closely associated with nucleotide metabolites.

Within the lipid class, phospholipid metabolism was significantly enriched (> tenfold) at all examined time points. This enrichment was associated with metabolites involved in the syntheses of phosphatidylcholine and phosphatidylethanolamine (PtdEth), which together constitute about 75–85% of phospholipids in the parasite [49]. Dihydroxyacetone phosphate (DHAP) was also associated with enrichment in this and other lipid subordinate pathways, except for that of linoleic acid metabolism, which does not involve DHAP. The enrichment in linoleic acid metabolism was associated with an increased abundance of PUFAs, such as arachidonate (20:4n6), linoleate (18:2n6), docosapentaenoate (22:5n3), and docosahexaenoate (22:6n3). All of these metabolites showed increased abundance in iRBC cultures relative to uRBC cultures (Additional file 6). In mammals, docosahexaenoate (22:6n3) and arachidonate (20:4n6) can be synthesized from α-linolenic acid (18:3n3) and linoleate (18:2n6), respectively [50], although synthesis of PUFAs has not previously been characterized in *P. falciparum*.

Overall, the enrichment in each metabolite subclass and associated metabolites showed characteristics of typical parasite development, where amino acids are used for protein synthesis, nucleotides are synthesized for DNA production, and lipids are synthesized for the development of lipid membranes. The next section describes the abundance of different phospholipid and fatty-acid metabolites in iRBC and uRBC cultures.

### Lipid metabolism in infected and uninfected cultures

**Fig. 5:**
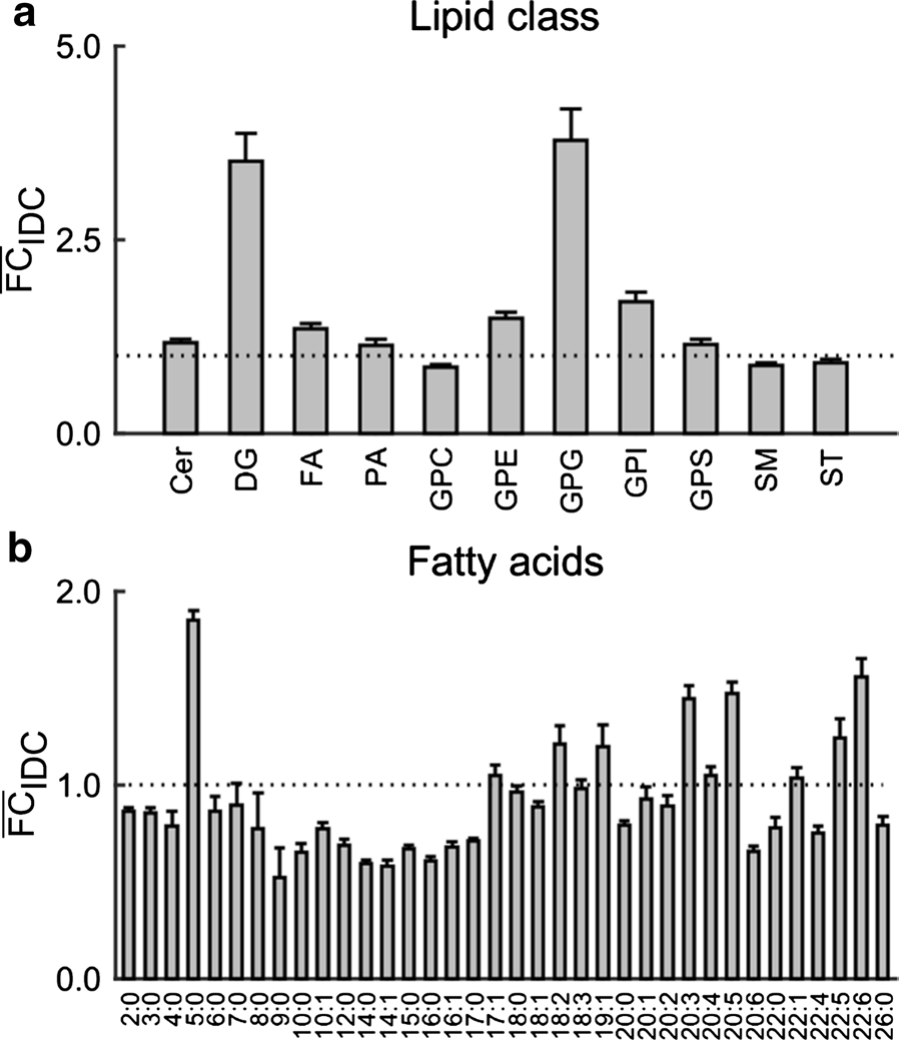
Fold change in abundance of lipid and fatty acid metabolites during the intraerythrocytic developmental cycle (IDC). **a** Lipid metabolites were classified according to the *LIPID MAPS Structure Database* [51] into 13 subordinate classes. The figure shows fold changes in lipid classes that contain two or more metabolites. The fold changes were greatest for diacylglycerol (DG) and glycerophosphoglycerol (GPG) metabolites. **b** Fold change in abundance of fatty acids based on different carbon-chain lengths. The fold change was greatest for the 5-carbon fatty acids (~ 1.8-fold in iRBC cultures relative to uRBC cultures), followed by a number of 18-carbon, 20-carbon, and 22-carbon polyunsaturated fatty acids (~ 1.5-fold). The $\overline{\text{FC}}_{\text{IDC }}$ was computed as the average ${\text{FC}}_{\text{IDC}}$ (described in Fig. 3c) when a metabolite class contained more than one metabolite. Each error bar shows the standard deviation of the ${\text{FC}}_{\text{IDC}}$ of metabolites present in a metabolite class. *Cer* ceramide, *DG* diacylglycerol, *FA* fatty acid amide, *PA* glycerophosphate, *GPC* glycerophosphocholine, *GPE* glycerophosphoethanolamine, *GPG* glycerophosphoglycerol, *GPI* glycerophosphoinositol, *GPS* glycerophosphoserine, *SM* phosphosphingolipid, *ST* sterol

Figure 5a shows the fold change in abundance of phospholipids, which are characterized based on the classification system of the *LIPID MAPS Structure Database* [51]. GPGs and diacylglycerols (DGs) showed the largest increase. Consistent with previous reports [52, 53], a roughly fourfold increase in DGs occurred during the IDC. DGs are used to synthesize triglycerides via diacylglycerol acyltransferase, an essential enzyme during the IDC [54]. In contrast, the increased abundance in GPGs was associated with 1-palmitoyl-GPG (16:0) and 1-stearoyl-GPG (18:0), which have not previously been characterized in *P. falciparum*. These LPGs are formed when phospholipase A2 hydrolyzes phosphatidylglycerol [55], and can be reacylated by the activity of acyl-CoA:lysophosphatidylglycerol acyltransferase to form phosphatidylglycerol. A BLAST-homology search of proteins homologous to human acyl-CoA:lysophosphatidylglycerol acyltransferase in the *P. falciparum* genome database [26] did not identify any candidate enzymes. This suggests that LPGs in *P. falciparum* may have some additional functions, such as cell proliferation [56], migration [57], cell adhesion [58], or Ca^2+^ signaling [59], which have been identified in mammalian cells.

Figure 5b shows the distribution of fatty acids with different carbon-chain lengths in iRBC cultures relative to the uRBC cultures. Apart from the increase seen in a 5-carbon fatty acid, five PUFA metabolites, i.e., docosahexaenoate (22:6n3), docosapentaenoate (22:5n6), docosapentaenoate (22:5n3), mead acid (20:3n9), and eicosapentaenoate (20:5n3), increased ~ 1.5-fold in iRBC cultures relative to uRBC cultures (see also Additional file 7: Sheet 2). These PUFAs cannot be synthesized in mature erythrocytes as they lack an essential enzyme necessary for fatty acid biosynthesis [60]. In contrast, *P. falciparum* should be able to synthesize PUFAs because it possesses the necessary elongases and desaturases [61, 62]. PUFAs are precursors of eicosanoids, which have immunosuppressive roles [63]. *P. falciparum*, under in vitro conditions, can produce eicosanoids when supplemented with a PUFA [64]. Eicosanoids also mediate fever and inflammation, and have numerous other functions [65]. These data show that the parasites, under in vitro conditions, can produce precursors that mediate inflammatory and immunosuppressive responses in an infected human host.

The lipids quantified here were further compared with those obtained from the D2 strain of *P. falciparum* in a lipidomics study by Gulati et al. [53], in which parasites were isolated from infected erythrocytes by saponin treatment prior to LC–MS measurements and a total of 304 lipid metabolites were measured during the IDC. Most of these lipid metabolites (239 of 304) had a carbon-chain length greater than 26, which was the maximum length in this study. In the current study, 17 (out of 65 possible) metabolites belonged to seven lipid subclasses. Following Gulati et al. [53], the abundance of a given lipid metabolite was normalized by the total lipid abundance at each time point, and then the Gulati et al. data were re-normalized to the 17 metabolites to make the datasets comparable. Table 3 lists the subclasses and their average fractions during the IDC as measured by both studies. Additional file 8 includes detailed comparisons of individual metabolites within each subclass. In agreement with the study by Gulati et al. [53], sphingomyelins—the lipid class with the third highest abundance levels in *P. falciparum* [53]—showed the highest abundance during the IDC (this study: 93.7% vs. Gulati et al.: 96.4%). Notably, the abundance fraction of the lyso phosphatidylinositol (PtdIns) class was lower in the current study than in the study by Gulati et al. [53]. Given the difference in the study design (purified parasites vs. co-culture), this discrepancy suggests that lyso PtdIns metabolites are primarily synthesized by *P. falciparum*. For other lipid subclasses, there was general agreement between the studies as their average fractions during the IDC were of the same order of magnitude. Furthermore, the novel lipid classes of dihydrosphingomyelin, lyso PtdEth, and lyso PtdIns characterized by Gulati et al. [53] were also present in the current study. The final section also provides details on the quantification of a novel lysophosphatidylglycerol subclass, which was uniquely present in iRBC cultures.

**Table 3:** Lipid metabolites quantified in this study and Gulati et al. [53]

Lipid subclass	N_metabolites_	Percentage of each lipid subclass, *f*_IDC_ (σ)	
		This study	Gulati et al.
Sphingomyelin	4	93.7 (0.61)	96.4 (0.50)
dhSM	2	4.19 (0.19)	1.36 (0.08)
Ceramide	2	0.64 (0.04)	0.37 (0.11)
LacCer	1	0.07 (0.01)	0.06 (0.02)
Lyso PtdCho	3	1.10 (0.82)	0.91 (0.19)
Lyso PtdEth	3	0.31 (0.03)	0.73 (0.23)
Lyso PtdIns	2	0.02 (0.01)	0.17 (0.06)

*N*_*metabolites*_ number of metabolites within each subclass, *f*_IDC_ average percentage of a subclass synthesized during the intraerythrocytic developmental cycle (IDC), *σ* standard deviation, *dhSM* dihydrosphingomyelin, *LacCer* lactosylceramide, *PtdCho* phosphatidylcholine, *PtdEth* phosphatidylethanolamine, *PtdIns* phosphatidylinositol

## Discussion

### Metabolic changes that characterize parasite development

**Fig. 6:**
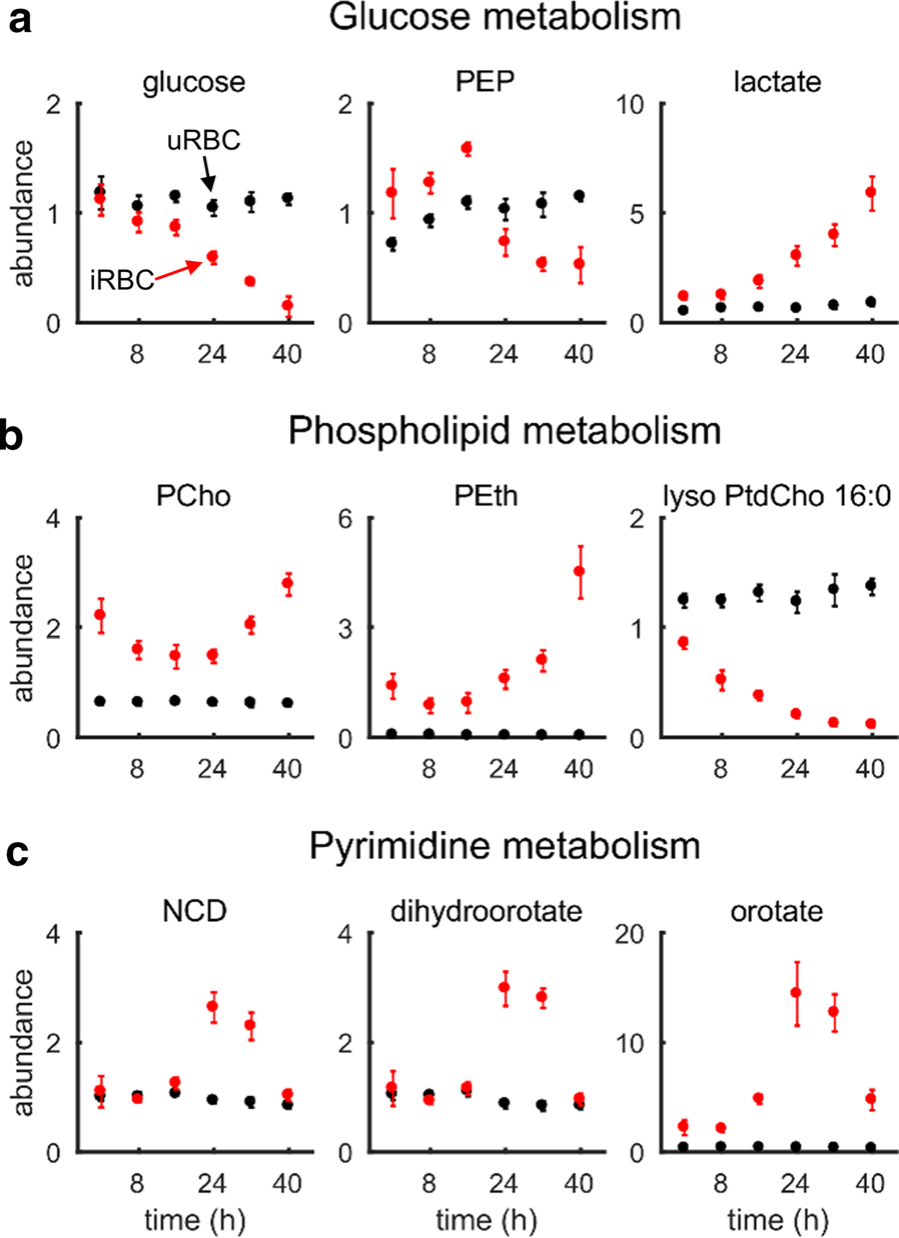
Normalized abundance of important metabolites of glucose, phospholipid, and pyrimidine metabolism in uninfected (uRBC) and parasite-infected erythrocyte (iRBC) cultures. **a** Abundance of glucose, phosphoenolpyruvate (PEP), and lactate during the intraerythrocytic developmental cycle (IDC). Glucose decreased in iRBC cultures, whereas it was stable in uRBC cultures. The increase in lactate was commensurate with glucose consumption, indicating active parasite metabolism. **b** Abundance of phosphocholine (PCho), phosphoethanolamine (PEth), and lyso phosphatidylcholine (PtdCho) 16:0 during the IDC. PCho and PEth are precursors of PtdCho and phosphatidylethanolamine, respectively, which account for ~ 75% to 85% of parasite phospholipids [49]. In addition to PEth, the parasite also utilizes lyso PtdCho to synthesize PtdCho [69], which also decreased over time in iRBC cultures. **c** Parasites synthesize *N*-carbamoyl-l-aspartate (NCD) in the first step, dihydroorotate in the second step, and orotate in the third step of de novo pyrimidine synthesis [27]. These metabolites increased in the iRBC cultures, consistent with the synthesis of parasite DNA [70]

The previous section described analyses of metabolic changes in lipid metabolism, which are associated with processes of the parasite that can modulate the host immune system [64, 66]. This section considers metabolic changes in iRBC and uRBC cultures that capture the development of the parasite during the IDC. Figure 6a shows three key metabolites of glucose metabolism in uRBC and iRBC cultures. Glucose remained stable in uRBC cultures, whereas it decreased roughly linearly in iRBC cultures [29], ultimately becoming exhausted at the end of the IDC. Concurrently with the ~ eightfold decrease of glucose in iRBC cultures at the 40-h time point relative to the 0-h time point, there was a ~ fivefold increase in lactate. These observations suggest that approximately 60% of the consumed glucose is oxidized to lactate in iRBC cultures, which is within the expected values (60–70%) reported in the literature [29].

Phosphoenolpyruvate (PEP), a glycolysis intermediate, also showed higher abundance in iRBC cultures relative to uRBC cultures early in the IDC (0–16 h), but only half the abundance in uRBC cultures later in the IDC (24–40 h). *Plasmodium falciparum* converts PEP into products, such as pyruvate [67] and oxaloacetate [68], which are important metabolites for pathways including the TCA cycle. The increased utilization of PEP at later time points suggests an increased use of TCA cycle reactions late in the IDC.

There was significant enrichment in phospholipid metabolites (Fig. 4) associated with the synthesis of PtdCho and PtdEth, which comprise up to 50% and 45%, respectively, of the total phospholipid content in purified parasites [49]. Figure 6b shows temporal variations in phosphocholine (PCho) and phosphoethanolamine (PEth), which are precursors of PtdCho and PtdEth, respectively, in the Kennedy pathway [71]. Both metabolites exhibited the highest abundance in iRBC cultures towards the end of the IDC, commensurate with the increased demand for membrane lipids in the developing merozoites. *P. falciparum* can also salvage host lyso PtdCho to synthesize PtdEth [69]. Consistent with these findings, lyso PtdCho decreased ~ eightfold in iRBC cultures at the 40-h time point relative to the 0-h time point.

The parasite also needs to synthesize purine and pyrimidine nucleotides for DNA synthesis. It does this by salvaging purine nucleotides from the host erythrocyte [72], and synthesizing pyrimidine nucleotides de novo [48]. Figure 6c depicts the temporal profiles of three pyrimidine metabolites, *N*-carbamoyl-l-aspartate, dihydroorotate, and orotate, which are synthesized in the first, second, and third steps of pyrimidine metabolism, respectively [27]. All three metabolites from iRBC cultures showed a temporal pattern consistent with *Plasmodium* DNA synthesis [70]. In contrast, these metabolites were stable in uRBC cultures because pyrimidine metabolites only exist in small concentrations in human erythrocytes [48]. Taken together, the pattern of metabolic changes in the iRBC shown in Fig. 6 reflect some processes necessary for parasite development during the IDC.

**Fig. 7:**
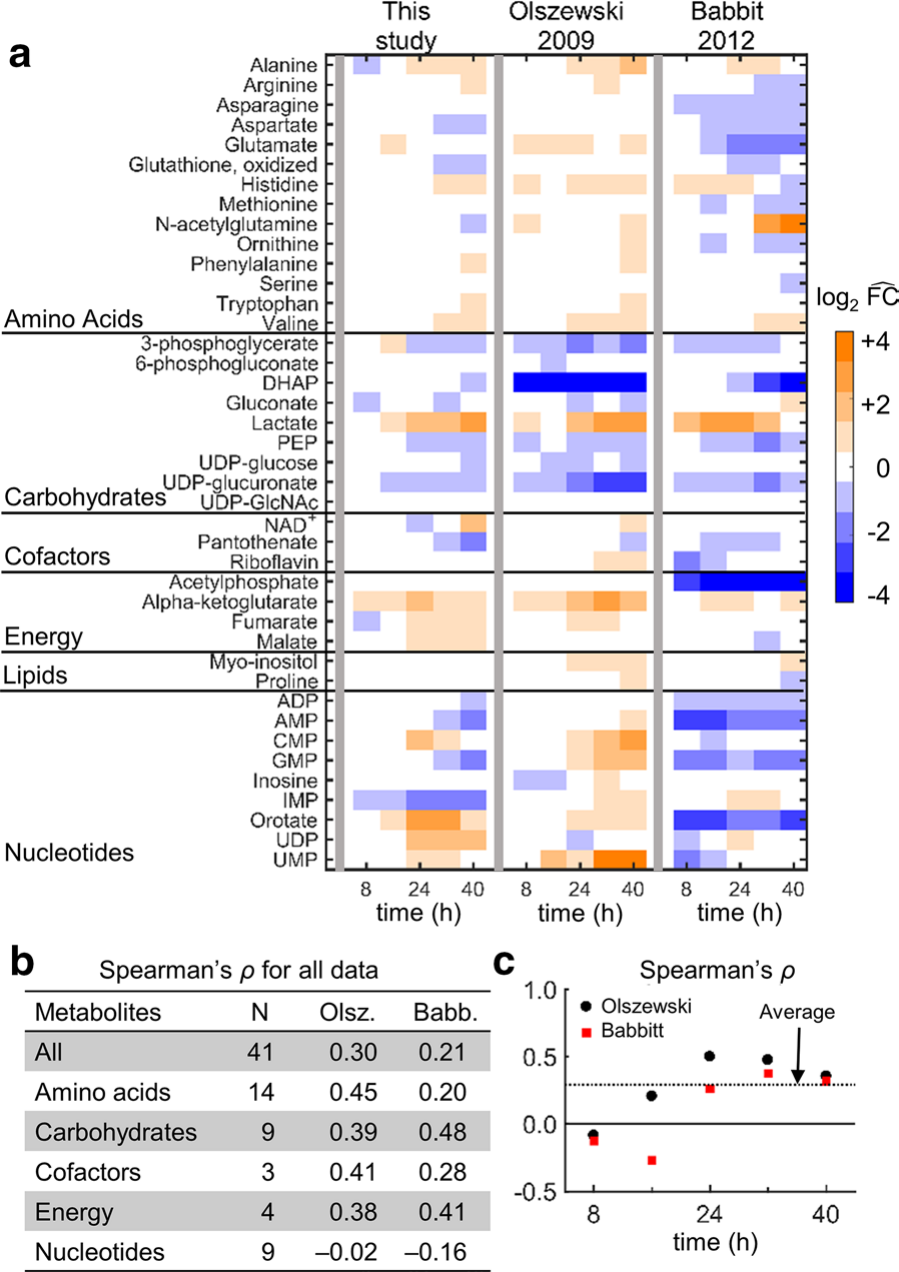
Metabolite and metabolic pathway concordance between this study and studies by Olszewski et al. [4] and Babbitt et al. [8]. **a** Temporal profiles of metabolite abundance quantified during the intraerythrocytic developmental cycle in the three studies. Metabolite abundances were normalized by their value at t = 0 h (grey vertical bar) to allow comparison across studies. Metabolites are grouped by metabolite class. **b** Spearman’s *ρ* computed for metabolites quantified in all three studies (N = 41) and within each metabolite class. The correlation for the lipid class, which contained only two metabolites, was not computed. **c** Spearman’s *ρ* computed for all metabolites at 8, 16, 24, 32, and 40 h. In comparisons with both studies, the correlation was near zero at the 8-h time point, i.e., when parasite metabolism is least active [73]. The dotted line shows the average (~ 0.3) of the correlations at each time point for both studies. *ADP* adenosine diphosphate, *AMP* adenosine monophosphate, *CMP* cytidine monophosphate, *DHAP* dihydroxyacetone phosphate, $\widehat{\text{FC}}$ metabolite abundance normalized with respect to t = 0 h, *GMP* guanosine monophosphate, *IMP* inosine monophosphate, *NAD*^*+*^ nicotinamide adenine dinucleotide (oxidized), *PEP* phosphoenolpyruvate, *UDP* uridine diphosphate, *UMP* uridine monophosphate

Next, the results of the current study were compared with metabolite changes in iRBC cultures measured in two previous independent studies. Figure 7a shows the abundance of metabolites (grouped by class) during the IDC. Carbohydrate metabolites, such as lactate and PEP, showed temporal variation in abundance similar to that reported by Olszewski et al. [4] and Babbitt et al. [8]. To quantify the degree of similarity between this study and each of these two studies, Spearman’s coefficient (*ρ*) was computed for metabolites belonging to each of the five different classes (Fig. 7b). The correlation between the data in this study and Olszewski’s data was highest for amino acid metabolites (0.45; *p* ≤ 0.01). In contrast, the same correlation between the data in this study and Babbitt’s data was only 0.21 (*p* = 0.06). To quantify the correlation between the three studies over the course of the IDC, Spearman’s *ρ* was computed between the results of this study and those of the other two studies at each time point. The correlations were lowest early in the IDC and increased at later time points. Ring-stage parasites are less metabolically active during early stages of IDC [73] and, hence, the low correlations observed at the 8-h time point could be attributed to experimental differences between the three studies rather than differences in parasite metabolism. In contrast, parasite metabolic activity is high during the intermediate and late stages of the IDC [73], which presumably results in more robust metabolite measurements and better correlations with the two studies at the 24-h time point and beyond.

### Metabolites uniquely associated with parasite infection

To identify infection-specific metabolite alterations at the early (0–8 h), intermediate (16–24 h), and late (32–40 h) stages of the IDC, the variation in abundance levels was examined as a function of the infection status of the culture and time. Separate 2 × 2 two-way ANOVAs conducted for each of the 501 metabolites at each stage (Additional file 9), with time point (0 and 8 h, 16 and 24 h, or 32 and 40 h) and infection status (infected and uninfected) as the between-subject factors, revealed that 42, 107, and 36 metabolites showed an infection-specific change at the early, intermediate, and late stages, respectively, as assessed by the significant interaction between time point and infection status (F_1,12_ values ≥ 4.78, ∀ *p *≤ 0.05; *q* < 0.10).

**Fig. 8:**
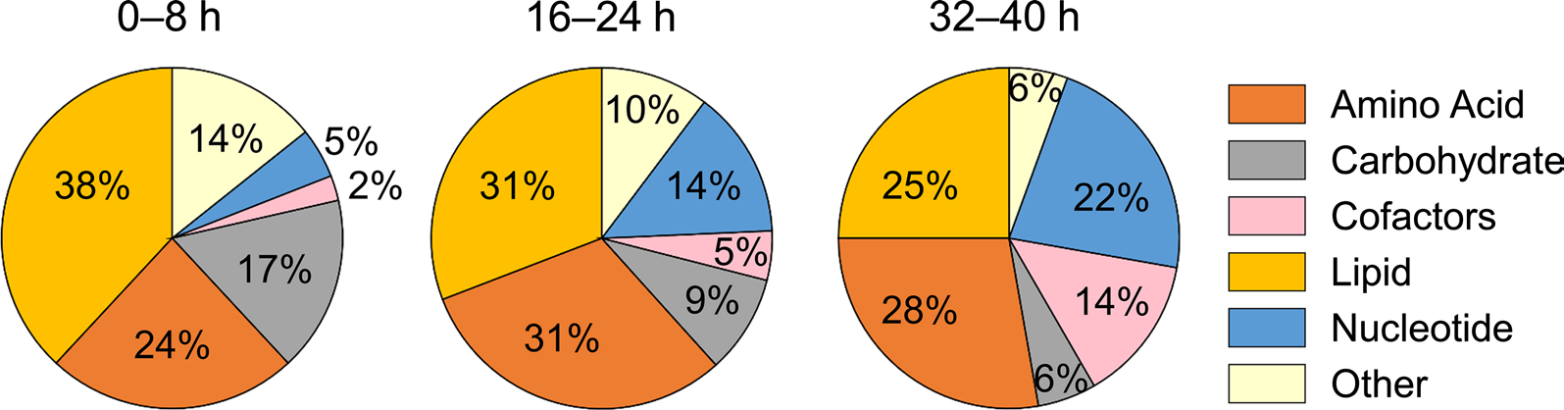
Distribution of significantly altered metabolites among major metabolic pathways at 0–8 h, 16–24 h, and 32–40 h. Significantly altered metabolites (*p* ≤ 0.05; *q* < 0.10) were identified by performing a two-way analysis of variance on the metabolomic data from the cell fractions of uninfected and infected cultures at the indicated time points. At the earliest time points (0–8 h) most of the significantly altered metabolites belonged to the lipid class, but at later time points (16–24 h and 32–40 h) both amino acid and lipid class metabolites were equally perturbed, commensurate with the stage-dependent development of parasite metabolism. The pathway labelled “Cofactors” corresponds to metabolites that belong to cofactor and vitamin metabolism. The pathway labelled “Other” includes metabolites that do not belong to any of the major pathways

Figure 8 shows the percentages of these significantly altered metabolites that were associated with one of the five major metabolic pathways or a pathway category designated “Other” (for metabolites that did not belong to any of the major pathways). Overall, there was a relative shift from lipid to amino acid metabolism during the progression of the IDC.

At the earliest time points (0–8 h), significant changes occurred most frequently for metabolites belonging to lipid metabolism pathways (16 of 42: 38%), six of which were categorized as sphingolipid metabolites. At the intermediate time points (16–24 h), the absolute number and relative fraction of lipids decreased (33 of 107: 31%) and the relative contribution of amino acid metabolism increased (33 of 107: 31%). The decrease in lipid metabolism was also associated with a change from sphingolipid to lysophospholipid metabolism, which included metabolites that appeared uniquely in iRBC cultures, such as 1-palmitoyl-GPG (16:0) and 1-stearoyl-GPG (18:0) (Table 4). Both of these, which belong to the LPG class, increased more than threefold in abundance during the IDC, suggesting that they are functionally important for parasite metabolism. In mammalian cells, LPGs are known to increase intracellular Ca^2+^ [74, 75], although the receptor that mediates this increase is not known [76].

**Table 4:** Metabolites uniquely detected in iRBC cultures and present at all the time points

Metabolite	FC_IDC_ (SD)^a^	Pathway	Mass (amu)^d^
*Amino acid metabolism*			
Putrescine	4.87 (0.83)	Polyamine	89.1
*N*^4^-acetylspermidine	2.15 (0.20)	Polyamine	188.2
*Cofactor and vitamin metabolism*			
Nicotinate ribonucleoside	4.73 (0.67)	Nicotinate and Nicotinamide	256.1
Nicotinate adenine dinucleotide	1.68 (0.16)	Nicotinate and Nicotinamide	663.1
*Lipid metabolism*			
1-stearoyl-GPG (18:0)	4.69 (0.81)	Lysophospholipid	511.3
1-palmitoyl-GPG (16:0)^b^	3.51 (0.50)	Lysophospholipid	483.3
Inositol 1-phosphate	2.58 (0.23)	Inositol	259.0
1-oleoyl-GPI (18:1)^b^	2.46 (0.27)	Lysophospholipid	597.3
Stearoyl-arachidonoyl-glycerol (18:0/20:4)^b,c^	1.68 (0.11)	Diacylglycerol	662.6
Palmitoyl-linolenoyl-glycerol (16:0/18:3)^b^	1.59 (0.20)	Diacylglycerol	608.5
1-arachidonoyl-GPI (20:4)^b^	1.51 (0.12)	Lysophospholipid	619.3
1-oleoyl-GPS (18:1)	1.49 (0.19)	Lysophospholipid	522.3
*Nucleotide metabolism*			
Thymidine	3.35 (0.48)	Pyrimidine	241.1
2′-*O*-methylcytidine	3.25 (0.47)	Pyrimidine	258.1
Guanosine	2.45 (0.41)	Purine	284.1
Pseudouridine	1.55 (0.18)	Pyrimidine	245.1
Uridine 5′-diphosphate	1.42 (0.20)	Pyrimidine	403.0
5-methyluridine	1.29 (0.19)	Pyrimidine	257.1
*Peptide*			
Histidylalanine	1.11 (0.11)	Dipeptide	227.1

*amu* atomic mass unit, *GPG* glycerophosphoglycerol, *GPI* glycerophosphoinositol, *GPS* glycerophosphoserine, *SD* standard deviation

^a^Fold-change (FC_IDC_) values based on average abundance of a metabolite during the IDC in iRBC relative to the uRBC cultures

^b^Metabolite identified based on m/z ratio alone with no external standard for validation

^c^m/z ratio appeared twice in the same platform, as it is a structural isomer of another compound in the METABOLON© spectral library

^d^Values provided by METABOLON©

Late in the IDC (32–40 h), the fraction of lipid metabolites remained similar to that at 16–24 h, although it now consisted of more phospholipids. In particular, two metabolites that also markedly increased in abundance in iRBC cultures relative to uRBC cultures (Additional file 6) were part of this set (i.e., PCho and PEth). These metabolites also appeared in the pathway enrichment analysis. Of these, PEth showed the highest abundance [~ 20-fold within this group (Additional file 5) and ~ 14-fold during the IDC (Table 2)].

The ANOVA analysis suggests a dynamic progression of metabolic activity in iRBCs, commensurate with highly regulated processes of parasite development and the shifting demands placed on parasite metabolism. This is reflected in changes in lipid metabolism that are apparently correlated with the parasite stage—a shifting emphasis from sphingolipids and lysophospholipids involved in cell signaling, to phospholipid metabolism related to de novo synthesis and the accumulation of infection-specific membrane components.

Table 4 lists the metabolites that were uniquely detected in iRBC cultures at all time points in all four replicates. The abundance of nicotinate ribonucleoside (NR) increased ~ fivefold in the iRBC cultures. During the IDC, NR increases up to ~ 15-fold in the extra-cellular medium of iRBC cultures, but is undetectable in uRBC cultures [18], confirming the observation that NR was specific to the iRBC cultures. *P. falciparum* encodes an enzyme that spontaneously synthesizes NR from nicotinate mononucleotide [27, 38]. Interestingly, nicotinate mononucleotide increased ~ 34-fold in iRBC cultures (Table 2). These results suggest that excessive glucose utilization in the iRBC cultures can cause accumulation of NR, which is then excreted to the extra-cellular medium [18]. As discussed above, nicotinate mononucleotide (Table 2) was associated with excessive glucose utilization. Therefore, NR has the potential to be a *P. falciparum*-specific blood marker of malaria infection, because these parasites are voracious consumers of glucose [29] and can cause hypoglycaemia in infected hosts [77, 78]. Additional file 10 includes a complete list of metabolites detected in iRBC and uRBC cultures, along with their raw counts during the IDC.

## Conclusions

High-resolution metabolomics was employed to quantify metabolic alterations in uninfected and parasite-infected erythrocyte cultures at multiple time points during the IDC. Analyses of the collected data quantified approximately fivefold more metabolites than previous studies of similar scope. Detailed analyses comparing infected and uninfected cultures, coupled with the time-course data, showed overall agreement with previous studies of *P. falciparum* blood-stage infection, but also revealed new insights. In particular, a new phospholipid class of LPG metabolites present only in parasite-infected cultures was identified. Although these metabolites modulate Ca^2+^ signaling in mammalian cells [59], their functional role in *P. falciparum* biology is unknown.

The relative abundance of these polyunsaturated fatty acids in the iRBC cultures increased. These metabolites are precursors for synthesizing eicosanoid-signaling molecules [79], which when released into the host blood plasma can modulate fever and inflammation under in vivo conditions [63]. They are also known to promote gametocytogenesis [80] and, thus, may play a role during the blood-stage development of *P. falciparum*.

The improved characterization and quantification of lipid metabolites reveals highly dynamic usage of phospholipids during the IDC. Specifically, the results suggest that sphingolipid and lysophospholipid metabolites are significantly utilized at early and intermediate stages of the IDC, whereas phospholipid metabolites dominate during the late stages. Overall, the metabolomic data presented here provide an opportunity for targeted discovery of metabolic functions and biology in *P. falciparum*.

## Supplementary information

**Additional file 1.** List of metabolites not annotated in KEGG and HMDB database.

**Additional file 2.** List of metabolites annotated in *Plasmodium* metabolite database.

**Additional file 3.** List of metabolites annotated in Malaria Parasite Metabolic Pathways database.

**Additional file 4.** Metabolites that have greater than twofold change in average abundance during the IDC.

**Additional file 5.** Fold change in abundance of metabolites listed in Additional file 4 at 0, 8, 16, 24, 32, and 40 h time point.

**Additional file 6.** Fold change in average abundance of all metabolites detected in uRBC and iRBC cultures during the IDC.

**Additional file 7.** Fold change in average abundance of lipids (Sheet 1) and fatty acids (Sheet 2) related to Fig. 5.

**Additional file 8.** Comparison of lipid metabolites from this study with Gulati et al. [53].

**Additional file 9.** Results of two-way ANOVA performed on data from uRBC and iRBC cultures obtained at 0–8 h (Sheet 1), 16–24 h (Sheet 2), and 32–40 h (Sheet 3).

**Additional file 10.** Raw metabolomic data from uRBC and iRBC cultures obtained in quadruplicate at 0, 8, 16, 24, 32, and 40 h time point.

## Data Availability

All data generated or analysed during this study are included in this published article and its additional files.

## Abbreviations

ADP: Adenosine diphosphate
AMP: Adenosine monophosphate
amu: Atomic mass unit
ANOVA: Analysis of variance
BCAA: Branched-chain amino acid
CMP: Cytidine monophosphate
CoA: Co-enzyme A
DG: Diacylglycerol
DHAP: Dihydroxyacetone phosphate
dhSM: Dihydrosphingomyelin
ESI: Electrospray ionization
FA: Fatty acid
GMP: Guanosine monophosphate
GPC: Glycerophosphocholine
GPE: Glycerophosphoethanolamine
GPG: Glycerophosphoglycerol
GPI: Glycerophosphoinositol
GPS: Glycerophosphoserine
HCA: Hierarchical clustering analysis
HILIC: Hydrophilic-interaction chromatography
HMDB: Human metabolome database
IDC: Intraerythrocytic developmental cycle
IMP: Inosine monophosphate
iRBC: Parasite-infected erythrocyte culture
KEGG: Kyoto encyclopedia of genes and genomes
LacCer: Lactosylceramide
LPG: Lysophosphatidylglycerol
MACS: Magnetically activated cell sorting
NAD^+^: Nicotinamide adenine dinucleotide (oxidized)
NADH: Nicotinamide adenine dinucleotide (reduced)
NCD: *N*-carbamoyl-l-aspartate
NR: Nicotinate ribonucleoside
PC1: First principal component
PC2: Second principal component
PC3: Third principal component
PCA: Principal component analysis
PCho: Phosphocholine
PEP: Phosphoenolpyruvate
PEth: Phosphoethanolamine
PPP: Pentose phosphate pathway
PtdCho: Phosphatidylcholine
PtdEth: Phosphatidylethanolamine
PtdIns: Phosphatidylinositol
PUFA: Polyunsaturated fatty acid
QSEA: Quantitative pathway enrichment analysis
RP: Reverse phase
RPMI: Roswell Park Memorial Institute
SD: Standard deviation
SMPDB: Small molecule pathway database
TCA: Tricarboxylic acid
UDP: Uridine diphosphate
UMP: Uridine monophosphate
UPLC: Ultrahigh-performance liquid chromatography
uRBC: Uninfected erythrocyte culture
WBC: White blood cell
WHO: World Health Organization
